# On the robustness of social norm elicitation

**DOI:** 10.1007/s40881-024-00178-2

**Published:** 2024-08-09

**Authors:** Christian König-Kersting

**Affiliations:** Department of Banking and Finance, University of Innsbruck, Universitätsstraße 15, 6020 Innsbruck, Austria

**Keywords:** Social norms, Incentives, Beliefs, Robustness, C72, C90, D90

## Abstract

We study the robustness of Krupka and Weber’s method (2013) for eliciting social norms. In two online experiments with more than 1200 participants on Amazon Mechanical Turk, we find that participants’ response patterns are invariant to differences in the salience of the monetarily incentivized coordination aspect. We further demonstrate that asking participants for their personal first- and second-order beliefs without monetary incentives results in qualitatively identical responses in the case that beliefs and social norms are well aligned. Overall, Krupka and Weber’s method produces remarkably robust response patterns.

**Supplementary Information:**

The online version contains supplementary material available at 10.1007/s40881-024-00178-2.

## Introduction

Krupka and Weber’s (2013, KW henceforth) method has been widely used to elicit social norm perception in economic experiments. The main feature of the elicitation method is that it incentivizes participants to form a belief about what the modal response of all study participants is to a given question. For example, in the experiment of the original article of KW, participants are incentivized to correctly guess the modal social appropriateness rating of a series of hypothetical dictator game decisions.

There have been numerous tests of the robustness of KW’s method: D’adda et al. (2016) demonstrate that it is robust to order effects. Castillo et al. (2022) show that norms elicited for behavior in a relatively complex game do not differ between players with different roles or different experiences in the game. They also demonstrate that players with different incentives and those with different social preferences do not report substantially different norms. Lane et al. (2023) study the effects of competing focal points – which are conceptually similar to what we will refer to as dissonance below – and find KW’s method to be largely robust to their presence. Relatedly, Fallucchi and Nosenzo (2022) report that KW’s method is robust to the influences of alternative salient focal points in the decision environment.

In this article, we report on two online experiments conducted on Amazon Mechanical Turk with more than 1200 participants which were designed to study the robustness of KW’s method for social norm elicitation. Specifically, we test how the salience of the coordination game aspect and the monetary incentives affect participants’ responses. We also compare responses elicited using their method to non-incentivized first- and second-order beliefs. Finally, we assess how attention to the instructions and task understanding affect participants’ responses. In our robustness tests, we stay close to the original implementation of KW’s method and carefully vary key elements of the instructions.

There are substantial differences in the implementation of KW’s method across different studies. Some implementations ask participants to state their personal belief, but incentivize them to report what they believe most others to believe (e.g., Erkut et al., 2015; Kimbrough & Vostroknutov, 2016; Krupka & Weber, 2013). That is, participants are asked to respond according to their *individual* belief about the actual appropriateness of some action, but are paid for indicating the *most common* response among all participants in the session or study. As one’s personal belief and one’s belief about what most others think about an issue do not necessarily have to coincide, this creates a dissonance or even conflict. For example, one might hold the personal belief that allocation mechanisms should be procedurally fair, i.e., give everyone the *same chance* of obtaining the better of multiple outcomes. Yet, the same person might hold the belief that most others will favor allocation mechanisms that establish outcome fairness, i.e., give everyone the *same outcome*. More generally, this can be thought of as a decision situation with competing focal points (cf. Lane et al., 2023).

Clearly, instructions which allow for multiple interpretations lead to more noisy responses and reduced data quality. In the case of the KW method, there are studies in which participants are constantly reminded of their incentives (e.g., Barr et al. 2018; Erkut et al., 2015; Fallucchi & Nosenzo, 2022; Zhang et al., 2024), and others, in which participants are never reminded of them after the initial instructions (e.g., Gächter et al., 2013, 2017; Abbink et al., 2017; Vesely and Klöckner 2017; Huber & Huber, 2020).^1^ If a dissonance exists between task description and incentivization, changing the salience of the incentives might affect participants’ responses. In further studies, the potential conflict between the individual’s belief and the most common response by others has been resolved by harmonizing the wording of the instructions (e.g., Heinicke et al., 2022; Kölle et al., 2020; Sass et al., 2018).

If participants’ responses are affected by details of the implementation, comparisons of results across studies become difficult, hindering the accumulation of knowledge. Therefore, in this study we test if there are differences in responses across different implementations. With four treatments, we systematically vary the salience of the conflict between task wording and financial incentives. We (i) replicate the original task wording of KW, (ii) increase the salience of the conflict between stated task and incentivization through constant reminders of the incentive structure on the decision pages, (iii) decrease the salience by never reminding participants of the incentives, and (iv) finally resolve the conflict by rephrasing the task. Note that we focus on testing if the dissonance we identified in the original instructions affect norm elicitation. The data we collected is not suitable to answer the question which side of the dissonance – personal beliefs or the perception of others’s beliefs – is more closely related to the actual social norm. We do not find significant differences between the different variations in task wording, indicating that the elicitation method is largely robust to adaptations of the instructions.

The defining feature that sets KW’s method for social norms elicitation apart from others, is its incorporation of monetary incentives. This not only makes the method attractive for economists, but ensures that revealing their true norm perception lies in participants’ interest. A recent study shows that – at least in situations where first-order beliefs and social norms are quite well aligned – the response patterns do not substantially differ between using KW’s elicitation method and a simpler approach of “just asking”, without dedicated monetary incentives (Heinicke et al., 2022). Relatedly, Vesely (2015) do not find significant differences between incentivized and non-incentivized social appropriateness ratings for *ultimatum game* behavior. To formally test for differences between incentivized and non-incentivized response patterns in the context of *dictator decisions*, which are more prevalent among the studies using KW’s method, we conduct two additional treatments in which we ask participants to state their first- and second-order beliefs about the appropriateness of the various dictator allocations. That is, we ask participants, without monetarily incentivizing them, to state (v) what they personally believe to be socially appropriate, and (vi) what they think most people would consider socially appropriate behavior. We do not find responses in the incentivized KW elicitation procedure to differ significantly from stated first- and second-order beliefs. Note, that we rely on appropriateness ratings for *hypothetical* dictator decisions (as in KW 2013) only and cannot be sure that the method yields equally robust results for incentivized dictator decisions or other tasks.

After conducting these treatments in a first experiment, we realized that more than half of our participants displayed poor task understanding as revealed by a post-task questionnaire asking them to recall the task they had been given and the monetary incentives that had been put in place (if any).^2^ In response, we conducted a second experiment, again encompassing all six treatments. We added the two post-task questions as mandatory comprehension checks to the pre-task instructions, enforcing correct responses. We find that these comprehension checks drastically improve post-task recall of task and incentives, but – strikingly – the results do not differ significantly between experiments 1 and 2. The response patterns also do not differ between those that appear to understand the task and those that do not, which can potentially be explained by the overlap between first-order beliefs and social norms. Throughout the paper we present data from all participants in both experiments and data from those participants that correctly answered the post-task questions side-by-side.

Both experiments and all treatments were specifically designed to represent the different variations found in past implementations of KW’s method and tease out differences in behavior of respondents recruited from the same population. Across all treatments and both experiments, it has become clear that KW’s method is remarkably robust and insensitive to variations in task wording and the salience of incentives. In our setting, where first-order beliefs and social norms are quite well aligned, the responses elicited using KW’s method also do not differ significantly from asking for participants’ beliefs directly, without monetary incentives.

## Design

The basic design of the experiment follows the “give”-framing of situation 1 in the first experiment reported in Krupka and Weber (2013). Participants in the experiment are asked to give appropriateness ratings for allocation decisions that a hypothetical dictator can take in a two-player dictator game. In the game, player A owns an endowment of $10, while player B does not own anything ($0). Player A can give any amount from $0 to $10 (in $1 increments) to player B. For each of the 11 possible options (“give $0” to “give $10”) we ask participants in our experiment to rate how socially appropriate the action is perceived to be. Each option is presented separately and their order is randomized on the individual level. That is, we do not show a sorted choice list.

In our first *Baseline* treatment, participants are asked to “indicate whether [they] believe choosing that option is very socially inappropriate, somewhat socially inappropriate, somewhat socially appropriate, or very socially appropriate”. In addition, participants are reminded “that [they] will earn money […] if [their] response to a randomly-selected question is the same as the most common response provided in today's session” (both quotes: Krupka & Weber, 2013, ESM, p. 7). We use the same wording as in the original experiment. The wording creates an obvious dissonance: Participants are asked to respond according to their individual belief about the actual appropriateness, but are paid for indicating the most common response among all participants. Note that an individual’s actual belief about the action’s appropriateness does not have to be in line with everyone else’s belief. Take, for example, a rich participant who might believe it to be socially appropriate to only take 3 for themselves, but give 7 to the other participant, while being convinced that most people would consider an equal split allocation to be the socially appropriate choice. Krupka and Weber (2013) present participants with a series of situations, but only remind the participants of their financial response motives in the first. Similarly, in treatment Baseline, participants are only reminded of their financial incentives on the first appropriateness rating they provide. The reminder is not shown for subsequent ratings. Baseline conceptually replicates KW’s original implementation.

We conduct three treatments aimed at identifying the sensitivity of the norm elicitation method to the two conflicting elements of the task description. In treatment *Always*, participants constantly, i.e., for each rating, see the reminder of their financial incentive to indicate the appropriateness rating that they believe most participants to select. This treatment is meant to reinforce the dissonance between stated task and financial incentives. In treatment *Never*, participants only learn about their financial incentive as part of the instructions, but are not reminded of it on the decision screen. Thus, this treatment deliberately attenuates the dissonance between task wording and monetary incentives. In treatment *No Conflict*, we modify the instructions such that they do not ask for the participants’ personal appropriateness ratings. Instead, we explicitly ask them to consider what most people think and select the appropriateness rating accordingly, which completely eliminates the conflict between the task and the monetary incentives put in place.

Furthermore, we conduct two treatments in which we remove monetary incentives and the coordination aspect. In *First*, we simply ask participants to give their individual appropriateness ratings. We avoid any references to other participants’ views or additional payments beyond a fixed compensation for participating in the study. This treatment is free of conflict and asks for individual, first-order beliefs. In *Second*, we ask participants to state the appropriateness rating which they believe most participants in the session would give. That is, rather than attempting to elicit the social norm using KW’s method, we ask participants for their first- and second-order belief directly. Table 1 shows an overview of all treatments.

**Table 1:** Treatment overview

		Task wording		
		Individual belief		Most people’s beliefs
Payment	Incentivized	Baseline		No conflict
		Always	Never	
	Non-incentivized	First		Second

To stay true to previous implementations of the method in the literature, we did not include any comprehension questions in the instructions of the first experiment. Instead, we asked participants two questions about their task and their monetary incentives as part of a *post-task* questionnaire. Only 52%, respectively 44%, of our participants answered these questions correctly (see details in Sect. 3). Acknowledging that the online setting may differ from a traditional laboratory environment in terms of participant dedication to the task, we opted to subsequently run a second experiment, involving an identical set of treatments. For this second experiment, we added the same two questions as comprehension questions to the pre-task instructions to force participants to engage more with the instructions and task description. Participants had to complete these questions correctly before continuing with the experiment. That is, in experiment 2, we asked the same questions in the pre-task and in the post-task questionnaire.

We recruited a total of 1228 participants from the USA on Amazon Mechanical Turk. Table A1 in the Electronic Supplementary Material (ESM) breaks down the number of participants by treatment and includes a small set of demographic variables. The average age of our participants is 37.5 years, 38.0% are female,^3^ and 2.2% report to have had some university-level education in economics. The experiment took approximately 6 min to complete and we paid a fixed amount of $0.75 and an additional bonus of $0.75 if a participant indicated the modal response in a randomly selected choice situation. In treatments *First* and *Second*, we only pay the fixed amount. On a per-hour basis, participants earned $9.03 on average. The experiment was programmed with oTree (Chen et al., 2016).

## Results

We first present results pertaining to task understanding, as it is the main reason for conducting two experiments. In a second step, we establish the baseline norm response pattern. Then, we compare responses in the treatments designed to reinforce, attenuate, and eliminate the dissonance in the instructions to this baseline. Finally, we compare responses to KW’s norm elicitation procedure to responses gathered by asking participants without using the element of coordination and monetary incentivization.

### Task understanding

Responses to our comprehension checks in the first experiment reveal that, across all treatments, only 52% of our participants were able to correctly recall whether their task was to give appropriateness ratings based on a) their own personal belief or based on b) what they thought most people would believe. Similarly, across all treatments, just 44% of our participants were able to correctly identify whether their payment for the study a) depended on their ability to anticipate what most people believe, b) depended on their own personal beliefs, or c) was independent of their responses.

In the second experiment, in which participants had to answer the questions twice, once as part of the pre-task instructions and once in the post-task questionnaire, we observe an increase in the number of correct responses. In the post-task questionnaire of the second set of treatments, 69% correctly understood the task and 69% correctly understood the incentives (again across all treatments). Table 2 gives an overview of the shares of participants that answered the questions correctly in each treatment. Note that with and without pre-task comprehension checks, No Conflict shows the highest task understanding rates among all treatments based on KW’s method. It appears that participants have a better grasp of their task when any potential dissonance between incentivization and actual task description is eliminated.

**Table 2:** Share of participants that correctly answered the comprehension checks

Treatment	Experiment 1	Experiment 2	*p*-value
Task			
Baseline	47.06%	62.38%	0.028
Always	51.89%	61.54%	0.158
Never	51.49%	67.65%	0.019
No conflict	62.96%	79.21%	0.009
First	49.51%	68.32%	0.006
Second	51.00%	75.76%	< 0.001
All pooled	52.42%	69.08%	< 0.001
Incentives			
Baseline	52.94%	70.30%	0.011
Always	44.34%	61.54%	0.013
Never	43.56%	68.63%	< 0.001
No conflict	38.39%	71.29%	< 0.001
First	43.69%	74.26%	< 0.001
Second	41.00%	68.69%	< 0.001
All pooled	44.03%	69.08%	< 0.001

Note: The table shows the share of participants that correctly answered the post-task comprehension questions. The top half shows data for the task question, the bottom half shows data for the incentives question. The last column shows the p-value for a test of proportions of the equality of the two shares

### Baseline

**Fig. 1:**
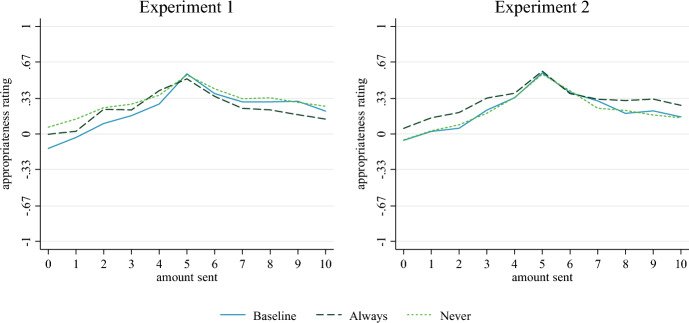
Mean appropriateness ratings in Baseline, Always, and Never

We observe the typical pattern of highest appropriateness ratings for 50/50 allocations between dictators and recipients, while lower and higher amounts transferred by the dictator are rated as less socially appropriate. The average ratings in our experiments lie in a relatively narrow band, ranging from −0.10 to 0.56 on the scale from −1 to 1.^4^ Figure 1 shows the average ratings for treatments *Baseline*, *Always*, and Never, separated by the first and second experiment. Differences between the two experiments are negligible (blue solid lines) for the Baseline treatment.

### Reinforcing the dissonance

In a first step, we compare responses in *Always* to those in *Baseline*. The only difference between these treatments is how prominently participants are reminded of their monetary incentives. In *Baseline* they are only reminded when giving the first rating; in *Always*, the reminder is constantly shown on the decision screen. As Fig. 1 suggests, there are no statistically significant differences in responses between the two treatments. This holds for both sets of treatments individually, as well as for the pooled data. Detailed test statistics of pairwise, two-sided Mann–Whitney-*U* tests for each allocation are reported in Table A2 in the ESM.

### Attenuating the dissonance

In a second step, we compare *Never* to *Baseline*. In *Never*, participants are informed about the monetary incentives as part of the instructions, but they are never reminded of these incentives when giving any of the appropriateness ratings. Compared to *Baseline*, the salience of the dissonance between task description and incentivization is reduced. The response patterns are shown in Fig. 1. We do not find systematic and significant differences between the responses in treatments *Baseline* and *Never*. This is true for both sets of treatments individually as well as the pooled data. Detailed test statistics are reported in Table A3 in the ESM.

### Eliminating the dissonance

**Fig. 2:**
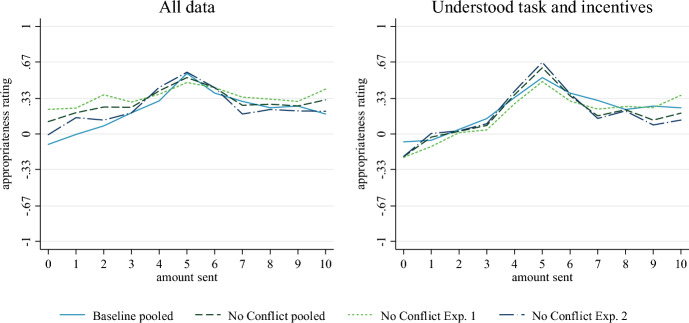
Mean appropriateness ratings in Baseline and No Conflict

In treatment *No Conflict* we modify the instructions to resolve the conflict between asking for personal opinions and paying for selecting the modal response. Participants are explicitly asked how most people would rate the allocations. Significant treatment differences (Mann––Whitney-*U* tests, *p* < 0.01) appear for small transfers ($0 to $3) in the first experiment and the pooled data, but not in the second experiment and for larger amounts (Fig. 2, left panel). If we restrict the data to participants who correctly answered the question on their incentives in the post-task questionnaire, the effects vanish (Fig. 2, right panel). Detailed test statistics are reported in Tables A4 and A5 in the ESM.

### Removing incentives

**Fig. 3:**
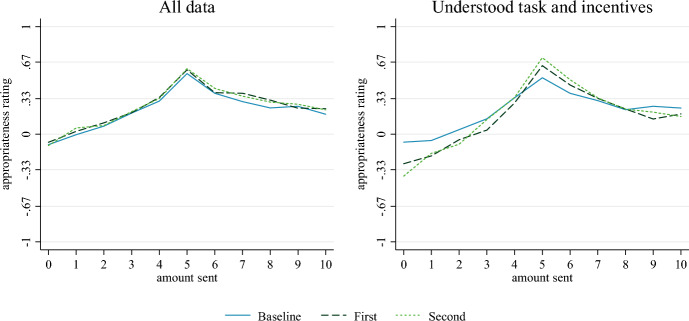
Mean appropriateness ratings in Baseline, First, and Second; pooled data

In treatments *First* and *Second*, we do not incentivize participants to select the appropriateness rating they think most other participants will select. Instead, we simply ask participants for their first- and second-order beliefs. That is, participants are asked, without incentivization, to rate the appropriateness of the different actions (*First*) and how they think most others would rate them (*Second*), respectively. The responses are depicted in Fig. 3. We find that aggregated responses elicited by asking for participants’ first- and second-order beliefs are practically identical to those elicited using KW’s incentivized elicitation method. In fact, we do not find any statistically significant differences between these treatments and any of the incentivized treatments. The results hold if we restrict the sample to participants who understood the incentives (or rather lack thereof). Details are reported in Tables A6 and A7 in the ESM.

## Discussion and conclusion

The most-widely used method of eliciting social norms in experiments appears to feature a dissonance between task wording and monetary incentives for the participant. The variety of implementations of KW’s task and the various adaptations of the original instructions highlight that it did not go unnoticed. In this study, we attempt to identify the effects this dissonance has on participants’ responses. We do not find any effects of reinforcing or attenuating the salience of monetary incentives relative to task wording. Even completely eliminating the dissonance by adapting the instructions does not affect participants’ behavior. The apparent dissonance is of no consequence.

In addition, we find that in our experiments, asking participants what they believe most others to think about the appropriateness of an action yielded the same pattern as implementing the social norm elicitation method based on coordination. While this finding resonates with recent work by Fallucchi et al. (2020) and Falk et al. (2018) who demonstrate that behavioral measures can be approximated by survey questions in the context of individuals’ willingness to compete and more general economic preferences, respectively, it comes with a caveat: In our setting, first-order beliefs and social norms, while different theoretical concepts, are closely aligned and may have considerable overlap. In contrast, we cannot expect the two methods to yield similar patterns in situations in which first-order beliefs and social norms are not as closely aligned. For example, Burks and Krupka (2012) as well as Krupka et al. (2022) demonstrate situations in which personal beliefs do not track social norms well. Thus, researchers selecting an elicitation method should closely examine their specific use case and make sure that the method they select maps well to the underlying construct.^5^

It is worthwhile to reconsider the observation that in our experiment there is hardly any difference in response patterns between the full sample of all participants and the restricted sample of participants who answered the comprehension check questions correctly. Given the previous discussion of the issue, this may well be a result of the close alignment between first-order beliefs and the social norm in the case of appropriateness ratings for dictator task allocations. It would be premature to conclude that task comprehension does not matter for social norm elicitation methods.

Conducting the experiment online, we find responses to lie within a relatively small range, resulting in distributions that are flatter than the ones typically found in other experiments implementing the method. We do not think that this is an effect of conducting the experiment online or with the Mechanical Turk sample, as Chang et al. (2019) and Fallucchi and Nosenzo (2022), for example, report larger ranges despite conducting their experiments online with a Mechanical Turk sample. We speculate that the smaller range is the result of presenting the individual allocations separately and in randomized order, rather than in a sorted choice list format. Nevertheless, conducting the experiment online and on a crowdworking platform on which participants may care less about the size of financial incentives than in laboratory environments is a limitation that should be kept in mind.

## Supplementary Information

Below is the link to the electronic supplementary material.

Supplementary file1 (DOCX 61 KB)

## Data Availability

The replication and supplementary materials for the studies are available at 10.17605/OSF.IO/6SAKB.

## Footnotes

1 Some studies adapted the wording, but do not fully resolve the dissonance between task and incentives. Notable examples are Chang et al. (2019) and D’Adda et al. (2016). There are also studies in which participants are not incentivized to select the same rating as most others, but are randomly matched in pairs for the purpose of payment determination (e.g., Barr et al. 2018).

2 Note that response patterns in the main KW task are quite similar in all treatments, irrespective of them being monetarily incentivized or not. Thus, it is unlikely that the poor task understanding revealed by the post-task questionnaire is purely the result of the questionnaire not being incentivized and participants considering this part less important.

3 One participant opted not to disclose their gender, 62.0% of our participants are male. For unknown reasons, there are more females in the second experiment than in the first: 35% vs. 41%, p = 0.03, two-sample test of proportions.

4 Other online and laboratory experiments typically report ratings ranging from −0.80 to 0.90 (cf. Krupka and Weber 2013, Erkut et al., 2015, Kimbrough and Vostruknutov 2016, Barr et al. 2018, Fallucchi and Nosenzo 2022).

5 One should also be aware that social norms can be subdivided into injunctive norms (perceptions about normatively appropriate behavior) and descriptive norms (prevalent or common behavior) (cf. Cialdini et al. 1990, 1991). These lead to further approaches for eliciting norms, such as the sequential matching task of Bicchieri and Xiao (2009) which has been used in a variety of contexts (e.g., Burstyn et al. 2020a, Burstyn et al. 2020b). The interested reader can find a comparison of the coordination-based method and the opinion-matching method in Lane et al. (2023).
