# Supplementary material for: On the robustness of social norm elicitation
Source: J Econ Sci Assoc. 2024 Aug 9;10(2):531–43. doi: 10.1007/s40881-024-00178-2 (PMC11967545; doi:10.1007/s40881-024-00178-2)
Supplement: Supplementary file 1 — Supplementary file1 (DOCX 61 KB) [file 40881_2024_178_MOESM1_ESM.docx]

# Electronic Supplementary Material

Table A1: Demographics

| Treatment | Observations | Age | Female | Economist |
| --- | --- | --- | --- | --- |
| *Experiment 1* |  |  |  |  |
| Baseline | 102 | 38.28 (11.09) | 41.18% | 1.96% |
| Always | 106 | 37.03 (10.86) | 43.40% | 2.83% |
| Never | 101 | 37.08 (10.24) | 32.67% | 1.98% |
| No Conflict | 108 | 38.55 (10.94) | 34.26% | 1.85% |
| First | 103 | 38.47 (11.50) | 29.13% | 3.88% |
| Second | 100 | 38.22 (11.01) | 29.00% | 3.00% |
|  |  |  |  |  |
| *Experiment 2* |  |  |  |  |
| Baseline | 101 | 36.68 (9.71) | 41.58% | 0.00% |
| Always | 104 | 37.60 (10.94) | 42.31% | 0.96% |
| Never | 102 | 36.72 (10.29) | 36.27% | 3.92% |
| No Conflict | 101 | 35.19 (10.35) | 46.53% | 1.98% |
| First | 101 | 38.90 (10.36) | 41.58% | 1.98% |
| Second | 99 | 37.26 (9.49) | 37.37% | 2.02% |

Note: The table shows the number of observations in each treatment, average age of the participants with standard deviation in parenthesis, the share of female participants, and the share of participants who reported to have had some formal training in economics on a university / college level.

Table A2: Appropriateness ratings *Baseline* vs. *Always*, all data

|  | *Baseline* | | | | | | | *Always* | | | | | | |  |
| --- | --- | --- | --- | --- | --- | --- | --- | --- | --- | --- | --- | --- | --- | --- | --- |
| action | mean | --- | -- | - | + | ++ | +++ | mean | --- | -- | - | + | ++ | +++ | p-value |
| *Exp 1* | *N=102* | | | | | | | *N=106* | | | | | | |  |
| send 0 | -0.13 | 0.25 | 0.16 | 0.12 | 0.23 | 0.17 | 0.08 | 0.00 | 0.25 | 0.09 | 0.09 | 0.19 | 0.25 | 0.11 | 0.188 |
| send 1 | -0.03 | 0.13 | 0.21 | 0.19 | 0.16 | 0.25 | 0.08 | 0.03 | 0.11 | 0.15 | 0.19 | 0.25 | 0.25 | 0.06 | 0.620 |
| send 2 | 0.10 | 0.01 | 0.26 | 0.16 | 0.21 | 0.28 | 0.08 | 0.23 | 0.04 | 0.14 | 0.13 | 0.29 | 0.26 | 0.13 | 0.153 |
| send 3 | 0.17 | 0.02 | 0.13 | 0.26 | 0.21 | 0.26 | 0.12 | 0.22 | 0.03 | 0.08 | 0.21 | 0.30 | 0.30 | 0.08 | 0.641 |
| send 4 | 0.28 | 0.02 | 0.05 | 0.22 | 0.31 | 0.31 | 0.09 | 0.40 | 0.02 | 0.03 | 0.09 | 0.39 | 0.39 | 0.08 | 0.106 |
| send 5 | 0.56 | 0.01 | 0.01 | 0.13 | 0.22 | 0.27 | 0.36 | 0.51 | 0.03 | 0.03 | 0.08 | 0.30 | 0.21 | 0.35 | 0.502 |
| send 6 | 0.38 | 0.01 | 0.05 | 0.16 | 0.34 | 0.25 | 0.19 | 0.35 | 0.04 | 0.07 | 0.12 | 0.30 | 0.30 | 0.17 | 0.946 |
| send 7 | 0.30 | 0.01 | 0.10 | 0.2 | 0.24 | 0.30 | 0.16 | 0.24 | 0.02 | 0.11 | 0.18 | 0.34 | 0.21 | 0.14 | 0.315 |
| send 8 | 0.30 | 0.04 | 0.09 | 0.16 | 0.25 | 0.29 | 0.17 | 0.22 | 0.03 | 0.12 | 0.21 | 0.21 | 0.30 | 0.13 | 0.402 |
| send 9 | 0.30 | 0.05 | 0.12 | 0.11 | 0.23 | 0.32 | 0.18 | 0.18 | 0.10 | 0.08 | 0.16 | 0.28 | 0.21 | 0.16 | 0.128 |
| send 10 | 0.21 | 0.11 | 0.13 | 0.12 | 0.19 | 0.24 | 0.23 | 0.14 | 0.16 | 0.09 | 0.10 | 0.23 | 0.28 | 0.13 | 0.303 |
|  |  |  |  |  |  |  |  |  |  |  |  |  |  |  |  |
| *Exp 2* | *N=101* | | | | | | | *N=104* | | | | | | |  |
| send 0 | -0.06 | 0.22 | 0.14 | 0.13 | 0.21 | 0.25 | 0.06 | 0.05 | 0.22 | 0.05 | 0.14 | 0.21 | 0.28 | 0.10 | 0.263 |
| send 1 | 0.02 | 0.17 | 0.16 | 0.11 | 0.23 | 0.24 | 0.10 | 0.15 | 0.12 | 0.12 | 0.13 | 0.26 | 0.22 | 0.15 | 0.195 |
| send 2 | 0.05 | 0.05 | 0.21 | 0.19 | 0.25 | 0.26 | 0.05 | 0.20 | 0.03 | 0.14 | 0.18 | 0.24 | 0.30 | 0.11 | 0.068 |
| send 3 | 0.22 | 0.03 | 0.05 | 0.25 | 0.33 | 0.27 | 0.08 | 0.33 | . | 0.06 | 0.21 | 0.28 | 0.31 | 0.14 | 0.105 |
| send 4 | 0.34 | 0.01 | 0.06 | 0.11 | 0.43 | 0.35 | 0.05 | 0.38 | . | 0.03 | 0.18 | 0.35 | 0.30 | 0.14 | 0.413 |
| send 5 | 0.57 | 0.01 | 0.02 | 0.07 | 0.27 | 0.33 | 0.31 | 0.58 | 0.01 | . | 0.10 | 0.25 | 0.31 | 0.34 | 0.751 |
| send 6 | 0.39 | 0.03 | 0.04 | 0.10 | 0.41 | 0.26 | 0.17 | 0.38 | 0.03 | 0.04 | 0.13 | 0.34 | 0.30 | 0.16 | 0.948 |
| send 7 | 0.31 | 0.03 | 0.10 | 0.13 | 0.28 | 0.34 | 0.13 | 0.32 | 0.04 | 0.09 | 0.12 | 0.33 | 0.25 | 0.18 | 0.881 |
| send 8 | 0.19 | 0.06 | 0.17 | 0.11 | 0.28 | 0.24 | 0.15 | 0.31 | 0.06 | 0.07 | 0.14 | 0.26 | 0.29 | 0.18 | 0.157 |
| send 9 | 0.21 | 0.09 | 0.11 | 0.13 | 0.26 | 0.25 | 0.17 | 0.32 | 0.06 | 0.12 | 0.10 | 0.22 | 0.28 | 0.23 | 0.173 |
| send 10 | 0.16 | 0.13 | 0.13 | 0.11 | 0.18 | 0.32 | 0.14 | 0.27 | 0.13 | 0.07 | 0.11 | 0.19 | 0.31 | 0.20 | 0.246 |
|  |  |  |  |  |  |  |  |  |  |  |  |  |  |  |  |
| *Pooled* | *N=203* | | | | | | | *N=210* | | | | | | |  |
| send 0 | -0.10 | 0.24 | 0.15 | 0.12 | 0.22 | 0.21 | 0.07 | 0.02 | 0.24 | 0.07 | 0.12 | 0.20 | 0.27 | 0.10 | 0.083 |
| send 1 | -0.00 | 0.15 | 0.18 | 0.15 | 0.19 | 0.24 | 0.09 | 0.09 | 0.11 | 0.13 | 0.16 | 0.25 | 0.23 | 0.10 | 0.209 |
| send 2 | 0.08 | 0.03 | 0.24 | 0.17 | 0.23 | 0.27 | 0.06 | 0.21 | 0.03 | 0.14 | 0.16 | 0.27 | 0.28 | 0.12 | 0.021 |
| send 3 | 0.20 | 0.02 | 0.09 | 0.26 | 0.27 | 0.27 | 0.10 | 0.28 | 0.01 | 0.07 | 0.21 | 0.29 | 0.30 | 0.11 | 0.148 |
| send 4 | 0.31 | 0.01 | 0.05 | 0.16 | 0.37 | 0.33 | 0.07 | 0.39 | 0.01 | 0.03 | 0.14 | 0.37 | 0.34 | 0.11 | 0.079 |
| send 5 | 0.56 | 0.01 | 0.01 | 0.10 | 0.24 | 0.30 | 0.33 | 0.55 | 0.02 | 0.01 | 0.09 | 0.28 | 0.26 | 0.34 | 0.774 |
| send 6 | 0.38 | 0.02 | 0.04 | 0.13 | 0.37 | 0.26 | 0.18 | 0.36 | 0.03 | 0.05 | 0.13 | 0.32 | 0.30 | 0.17 | 0.992 |
| send 7 | 0.30 | 0.02 | 0.10 | 0.16 | 0.26 | 0.32 | 0.14 | 0.28 | 0.03 | 0.10 | 0.15 | 0.33 | 0.23 | 0.16 | 0.529 |
| send 8 | 0.24 | 0.05 | 0.13 | 0.13 | 0.27 | 0.27 | 0.16 | 0.27 | 0.04 | 0.10 | 0.18 | 0.23 | 0.30 | 0.16 | 0.654 |
| send 9 | 0.26 | 0.07 | 0.11 | 0.12 | 0.24 | 0.29 | 0.17 | 0.25 | 0.08 | 0.10 | 0.13 | 0.25 | 0.24 | 0.20 | 0.925 |
| send 10 | 0.19 | 0.12 | 0.13 | 0.11 | 0.18 | 0.28 | 0.18 | 0.20 | 0.14 | 0.08 | 0.10 | 0.21 | 0.30 | 0.17 | 0.955 |

The table consists of three panels. The first shows data from the first experiment, the second shows data from the second, and the third presents pooled data. In each panel, the shares of participants that gave the respective rating for each amount sent by the dictator is reported. The modal response is highlighted. In addition, the mean appropriateness rating on a scale from -1 to 1 is given. The last column presents p-values of Mann-Whitney-U tests between the two treatments.

Table A3: Appropriateness ratings *Baseline* vs. *Never*, all data

|  | *Baseline* | | | | | | | *Never* | | | | | | |  |
| --- | --- | --- | --- | --- | --- | --- | --- | --- | --- | --- | --- | --- | --- | --- | --- |
| action | mean | --- | -- | - | + | ++ | +++ | mean | --- | -- | - | + | ++ | +++ | p-value |
| *Exp 1* | *N=102* | | | | | | | *N=101* | | | | | | |  |
| send 0 | -0.13 | 0.25 | 0.16 | 0.12 | 0.23 | 0.17 | 0.08 | 0.06 | 0.2 | 0.12 | 0.13 | 0.13 | 0.26 | 0.17 | 0.032* |
| send 1 | -0.03 | 0.13 | 0.21 | 0.19 | 0.16 | 0.25 | 0.08 | 0.14 | 0.11 | 0.14 | 0.16 | 0.21 | 0.19 | 0.20 | 0.074 |
| send 2 | 0.10 | 0.01 | 0.26 | 0.16 | 0.21 | 0.28 | 0.08 | 0.24 | 0.04 | 0.12 | 0.18 | 0.24 | 0.25 | 0.18 | 0.073 |
| send 3 | 0.17 | 0.02 | 0.13 | 0.26 | 0.21 | 0.26 | 0.12 | 0.28 | 0.05 | 0.09 | 0.18 | 0.24 | 0.24 | 0.21 | 0.175 |
| send 4 | 0.28 | 0.02 | 0.05 | 0.22 | 0.31 | 0.31 | 0.09 | 0.36 | 0.01 | 0.04 | 0.19 | 0.30 | 0.32 | 0.15 | 0.228 |
| send 5 | 0.56 | 0.01 | 0.01 | 0.13 | 0.22 | 0.27 | 0.36 | 0.55 | 0.01 | 0.04 | 0.12 | 0.17 | 0.29 | 0.38 | 0.898 |
| send 6 | 0.38 | 0.01 | 0.05 | 0.16 | 0.34 | 0.25 | 0.19 | 0.42 | 0.01 | 0.06 | 0.15 | 0.21 | 0.38 | 0.20 | 0.300 |
| send 7 | 0.30 | 0.01 | 0.10 | 0.20 | 0.24 | 0.30 | 0.16 | 0.33 | 0.02 | 0.09 | 0.18 | 0.21 | 0.33 | 0.18 | 0.617 |
| send 8 | 0.30 | 0.04 | 0.09 | 0.16 | 0.25 | 0.29 | 0.17 | 0.34 | 0.02 | 0.13 | 0.12 | 0.27 | 0.22 | 0.25 | 0.578 |
| send 9 | 0.30 | 0.05 | 0.12 | 0.11 | 0.23 | 0.32 | 0.18 | 0.29 | 0.05 | 0.12 | 0.12 | 0.24 | 0.28 | 0.20 | 0.925 |
| send 10 | 0.21 | 0.11 | 0.13 | 0.12 | 0.19 | 0.24 | 0.23 | 0.26 | 0.13 | 0.07 | 0.1 | 0.24 | 0.24 | 0.23 | 0.762 |
|  |  |  |  |  |  |  |  |  |  |  |  |  |  |  |  |
| *Exp 2* | *N=101* | | | | | | | *N=102* | | | | | | |  |
| send 0 | -0.06 | 0.22 | 0.14 | 0.13 | 0.21 | 0.25 | 0.06 | -0.06 | 0.26 | 0.09 | 0.13 | 0.23 | 0.18 | 0.12 | 0.979 |
| send 1 | 0.02 | 0.17 | 0.16 | 0.11 | 0.23 | 0.24 | 0.10 | 0.03 | 0.16 | 0.13 | 0.17 | 0.23 | 0.22 | 0.11 | 0.932 |
| send 2 | 0.05 | 0.05 | 0.21 | 0.19 | 0.25 | 0.26 | 0.05 | 0.09 | 0.08 | 0.10 | 0.23 | 0.33 | 0.22 | 0.05 | 0.780 |
| send 3 | 0.22 | 0.03 | 0.05 | 0.25 | 0.33 | 0.27 | 0.08 | 0.19 | 0.03 | 0.08 | 0.24 | 0.33 | 0.25 | 0.08 | 0.685 |
| send 4 | 0.34 | 0.01 | 0.06 | 0.11 | 0.43 | 0.35 | 0.05 | 0.34 | 0.02 | 0.06 | 0.14 | 0.37 | 0.27 | 0.14 | 0.750 |
| send 5 | 0.57 | 0.01 | 0.02 | 0.07 | 0.27 | 0.33 | 0.31 | 0.56 | 0.02 | 0.02 | 0.06 | 0.29 | 0.29 | 0.31 | 0.879 |
| send 6 | 0.39 | 0.03 | 0.04 | 0.10 | 0.41 | 0.26 | 0.17 | 0.40 | 0.02 | 0.05 | 0.09 | 0.37 | 0.33 | 0.14 | 0.766 |
| send 7 | 0.31 | 0.03 | 0.10 | 0.13 | 0.28 | 0.34 | 0.13 | 0.24 | 0.03 | 0.07 | 0.23 | 0.30 | 0.25 | 0.12 | 0.299 |
| send 8 | 0.19 | 0.06 | 0.17 | 0.11 | 0.28 | 0.24 | 0.15 | 0.22 | 0.05 | 0.14 | 0.14 | 0.28 | 0.25 | 0.15 | 0.787 |
| send 9 | 0.21 | 0.09 | 0.11 | 0.13 | 0.26 | 0.25 | 0.17 | 0.18 | 0.10 | 0.16 | 0.10 | 0.25 | 0.20 | 0.20 | 0.746 |
| send 10 | 0.16 | 0.13 | 0.13 | 0.11 | 0.18 | 0.32 | 0.14 | 0.15 | 0.17 | 0.03 | 0.19 | 0.21 | 0.25 | 0.17 | 0.914 |
|  |  |  |  |  |  |  |  |  |  |  |  |  |  |  |  |
| *Pooled* | *N=203* | | | | | | | *N=203* | | | | | | |  |
| send 0 | -0.10 | 0.24 | 0.15 | 0.12 | 0.22 | 0.21 | 0.07 | 0.00 | 0.23 | 0.10 | 0.13 | 0.18 | 0.22 | 0.14 | 0.126 |
| send 1 | -0.00 | 0.15 | 0.18 | 0.15 | 0.19 | 0.24 | 0.09 | 0.08 | 0.13 | 0.13 | 0.16 | 0.22 | 0.20 | 0.15 | 0.187 |
| send 2 | 0.08 | 0.03 | 0.24 | 0.17 | 0.23 | 0.27 | 0.06 | 0.16 | 0.06 | 0.11 | 0.20 | 0.29 | 0.23 | 0.11 | 0.147 |
| send 3 | 0.20 | 0.02 | 0.09 | 0.26 | 0.27 | 0.27 | 0.10 | 0.24 | 0.04 | 0.08 | 0.21 | 0.29 | 0.24 | 0.14 | 0.451 |
| send 4 | 0.31 | 0.01 | 0.05 | 0.16 | 0.37 | 0.33 | 0.07 | 0.35 | 0.01 | 0.05 | 0.16 | 0.33 | 0.30 | 0.14 | 0.270 |
| send 5 | 0.56 | 0.01 | 0.01 | 0.10 | 0.24 | 0.30 | 0.33 | 0.55 | 0.01 | 0.03 | 0.09 | 0.23 | 0.29 | 0.34 | 0.992 |
| send 6 | 0.38 | 0.02 | 0.04 | 0.13 | 0.37 | 0.26 | 0.18 | 0.41 | 0.01 | 0.05 | 0.12 | 0.29 | 0.35 | 0.17 | 0.332 |
| send 7 | 0.30 | 0.02 | 0.1 | 0.16 | 0.26 | 0.32 | 0.14 | 0.28 | 0.02 | 0.08 | 0.20 | 0.26 | 0.29 | 0.15 | 0.734 |
| send 8 | 0.24 | 0.05 | 0.13 | 0.13 | 0.27 | 0.27 | 0.16 | 0.28 | 0.03 | 0.13 | 0.13 | 0.28 | 0.23 | 0.20 | 0.588 |
| send 9 | 0.26 | 0.07 | 0.11 | 0.12 | 0.24 | 0.29 | 0.17 | 0.23 | 0.07 | 0.14 | 0.11 | 0.25 | 0.24 | 0.20 | 0.769 |
| send 10 | 0.19 | 0.12 | 0.13 | 0.11 | 0.18 | 0.28 | 0.18 | 0.20 | 0.15 | 0.05 | 0.14 | 0.22 | 0.24 | 0.20 | 0.884 |

The table consists of three panels. The first shows data from the first experiment, the second shows data from the second, and the third presents pooled data. In each panel, the shares of participants that gave the respective rating for each amount sent by the dictator is reported. The modal response is highlighted. In addition, the mean appropriateness rating on a scale from -1 to 1 is given. The last column presents p-values of Mann-Whitney-U tests between the two treatments, * denotes significance at 5%.

Table A4: Appropriateness ratings *Baseline* vs. *No Conflict*, all data

|  | *Baseline* | | | | | | | *No Conflict* | | | | | | |  |
| --- | --- | --- | --- | --- | --- | --- | --- | --- | --- | --- | --- | --- | --- | --- | --- |
| action | mean | --- | -- | - | + | ++ | +++ | mean | --- | -- | - | + | ++ | +++ | p-value |
| *Exp 1* | *N=102* | | | | | | | *N=108* | | | | | | |  |
| send 0 | -0.13 | 0.25 | 0.16 | 0.12 | 0.23 | 0.17 | 0.08 | 0.23 | 0.12 | 0.04 | 0.13 | 0.30 | 0.30 | 0.12 | <0.001*** |
| send 1 | -0.03 | 0.13 | 0.21 | 0.19 | 0.16 | 0.25 | 0.08 | 0.24 | 0.07 | 0.09 | 0.11 | 0.32 | 0.28 | 0.12 | 0.007** |
| send 2 | 0.10 | 0.01 | 0.26 | 0.16 | 0.21 | 0.28 | 0.08 | 0.36 | 0.03 | 0.09 | 0.11 | 0.27 | 0.30 | 0.20 | 0.002*** |
| send 3 | 0.17 | 0.02 | 0.13 | 0.26 | 0.21 | 0.26 | 0.12 | 0.30 | 0.01 | 0.08 | 0.19 | 0.31 | 0.25 | 0.16 | 0.163 |
| send 4 | 0.28 | 0.02 | 0.05 | 0.22 | 0.31 | 0.31 | 0.09 | 0.37 | 0.01 | 0.02 | 0.14 | 0.50 | 0.19 | 0.15 | 0.472 |
| send 5 | 0.56 | 0.01 | 0.01 | 0.13 | 0.22 | 0.27 | 0.36 | 0.48 | 0.02 | 0.03 | 0.06 | 0.36 | 0.33 | 0.19 | 0.047 |
| send 6 | 0.38 | 0.01 | 0.05 | 0.16 | 0.34 | 0.25 | 0.19 | 0.43 | 0.01 | 0.06 | 0.09 | 0.37 | 0.21 | 0.25 | 0.417 |
| send 7 | 0.30 | 0.01 | 0.10 | 0.20 | 0.24 | 0.30 | 0.16 | 0.34 | 0.05 | 0.05 | 0.13 | 0.31 | 0.31 | 0.15 | 0.671 |
| send 8 | 0.30 | 0.04 | 0.09 | 0.16 | 0.25 | 0.29 | 0.17 | 0.32 | 0.03 | 0.08 | 0.13 | 0.33 | 0.26 | 0.17 | 0.956 |
| send 9 | 0.30 | 0.05 | 0.12 | 0.11 | 0.23 | 0.32 | 0.18 | 0.30 | 0.06 | 0.08 | 0.13 | 0.24 | 0.29 | 0.19 | 0.993 |
| send 10 | 0.21 | 0.11 | 0.13 | 0.12 | 0.19 | 0.24 | 0.23 | 0.42 | 0.06 | 0.02 | 0.08 | 0.31 | 0.36 | 0.17 | 0.149 |
|  |  |  |  |  |  |  |  |  |  |  |  |  |  |  |  |
| *Exp 2* | *N=101* | | | | | | | *N=101* | | | | | | |  |
| send 0 | -0.06 | 0.22 | 0.14 | 0.13 | 0.21 | 0.25 | 0.06 | -0.01 | 0.30 | 0.04 | 0.10 | 0.22 | 0.21 | 0.14 | 0.651 |
| send 1 | 0.02 | 0.17 | 0.16 | 0.11 | 0.23 | 0.24 | 0.10 | 0.15 | 0.15 | 0.12 | 0.07 | 0.24 | 0.31 | 0.12 | 0.193 |
| send 2 | 0.05 | 0.05 | 0.21 | 0.19 | 0.25 | 0.26 | 0.05 | 0.13 | 0.09 | 0.15 | 0.15 | 0.26 | 0.23 | 0.13 | 0.361 |
| send 3 | 0.22 | 0.03 | 0.05 | 0.25 | 0.33 | 0.27 | 0.08 | 0.20 | 0.03 | 0.06 | 0.29 | 0.27 | 0.26 | 0.10 | 0.813 |
| send 4 | 0.34 | 0.01 | 0.06 | 0.11 | 0.43 | 0.35 | 0.05 | 0.43 | . | 0.02 | 0.12 | 0.34 | 0.46 | 0.07 | 0.072 |
| send 5 | 0.57 | 0.01 | 0.02 | 0.07 | 0.27 | 0.33 | 0.31 | 0.57 | 0.01 | 0.01 | 0.08 | 0.28 | 0.30 | 0.33 | 0.923 |
| send 6 | 0.39 | 0.03 | 0.04 | 0.10 | 0.41 | 0.26 | 0.17 | 0.43 | . | 0.02 | 0.18 | 0.22 | 0.47 | 0.12 | 0.318 |
| send 7 | 0.31 | 0.03 | 0.10 | 0.13 | 0.28 | 0.34 | 0.13 | 0.19 | 0.02 | 0.11 | 0.25 | 0.28 | 0.24 | 0.11 | 0.103 |
| send 8 | 0.19 | 0.06 | 0.17 | 0.11 | 0.28 | 0.24 | 0.15 | 0.23 | 0.04 | 0.13 | 0.14 | 0.34 | 0.21 | 0.15 | 0.829 |
| send 9 | 0.21 | 0.09 | 0.11 | 0.13 | 0.26 | 0.25 | 0.17 | 0.21 | 0.11 | 0.10 | 0.08 | 0.30 | 0.30 | 0.12 | 0.884 |
| send 10 | 0.16 | 0.13 | 0.13 | 0.11 | 0.18 | 0.32 | 0.14 | 0.21 | 0.14 | 0.06 | 0.08 | 0.35 | 0.23 | 0.15 | 0.975 |
|  |  |  |  |  |  |  |  |  |  |  |  |  |  |  |  |
| *Pooled* | *N=203* | | | | | | | *N=209* | | | | | | |  |
| send 0 | -0.10 | 0.24 | 0.15 | 0.12 | 0.22 | 0.21 | 0.07 | 0.12 | 0.21 | 0.04 | 0.11 | 0.26 | 0.25 | 0.13 | 0.004*** |
| send 1 | -0.00 | 0.15 | 0.18 | 0.15 | 0.19 | 0.24 | 0.09 | 0.20 | 0.11 | 0.11 | 0.09 | 0.28 | 0.29 | 0.12 | 0.006** |
| send 2 | 0.08 | 0.03 | 0.24 | 0.17 | 0.23 | 0.27 | 0.06 | 0.25 | 0.06 | 0.12 | 0.13 | 0.26 | 0.26 | 0.17 | 0.003*** |
| send 3 | 0.20 | 0.02 | 0.09 | 0.26 | 0.27 | 0.27 | 0.10 | 0.25 | 0.02 | 0.07 | 0.24 | 0.29 | 0.25 | 0.13 | 0.369 |
| send 4 | 0.31 | 0.01 | 0.05 | 0.16 | 0.37 | 0.33 | 0.07 | 0.40 | 0.00 | 0.02 | 0.13 | 0.42 | 0.32 | 0.11 | 0.106 |
| send 5 | 0.56 | 0.01 | 0.01 | 0.10 | 0.24 | 0.30 | 0.33 | 0.52 | 0.01 | 0.02 | 0.07 | 0.32 | 0.32 | 0.26 | 0.160 |
| send 6 | 0.38 | 0.02 | 0.04 | 0.13 | 0.37 | 0.26 | 0.18 | 0.43 | 0.00 | 0.04 | 0.13 | 0.30 | 0.33 | 0.19 | 0.212 |
| send 7 | 0.30 | 0.02 | 0.1 | 0.16 | 0.26 | 0.32 | 0.14 | 0.27 | 0.03 | 0.08 | 0.19 | 0.30 | 0.28 | 0.13 | 0.417 |
| send 8 | 0.24 | 0.05 | 0.13 | 0.13 | 0.27 | 0.27 | 0.16 | 0.28 | 0.03 | 0.11 | 0.13 | 0.33 | 0.23 | 0.16 | 0.838 |
| send 9 | 0.26 | 0.07 | 0.11 | 0.12 | 0.24 | 0.29 | 0.17 | 0.26 | 0.09 | 0.09 | 0.11 | 0.27 | 0.29 | 0.16 | 0.908 |
| send 10 | 0.19 | 0.12 | 0.13 | 0.11 | 0.18 | 0.28 | 0.18 | 0.32 | 0.10 | 0.04 | 0.08 | 0.33 | 0.30 | 0.16 | 0.261 |

The table consists of three panels. The first shows data from the first experiment, the second shows data from the second, and the third presents pooled data. In each panel, the shares of participants that gave the respective rating for each amount sent by the dictator is reported. The modal response is highlighted. In addition, the mean appropriateness rating on a scale from -1 to 1 is given. The last column presents p-values of Mann-Whitney-U tests between the two treatments, *** / ** / * denote significance at 0.5% / 1% / 5%.

Table A5: Appropriateness ratings *Baseline* vs. *No Conflict*, understood incentives only

|  | *Baseline* | | | | | | | *No Conflict* | | | | | | |  |
| --- | --- | --- | --- | --- | --- | --- | --- | --- | --- | --- | --- | --- | --- | --- | --- |
| action | mean | --- | -- | - | + | ++ | +++ | mean | --- | -- | - | + | ++ | +++ | p-value |
| *Exp 1* | *N=54* | | | | | | | *N=42* | | | | | | |  |
| send 0 | -0.23 | 0.33 | 0.15 | 0.09 | 0.19 | 0.22 | 0.02 | -0.05 | 0.26 | 0.05 | 0.12 | 0.31 | 0.24 | 0.02 | 0.277 |
| send 1 | -0.09 | 0.22 | 0.17 | 0.13 | 0.17 | 0.26 | 0.06 | -0.04 | 0.14 | 0.19 | 0.14 | 0.29 | 0.17 | 0.07 | 0.784 |
| send 2 | 0.06 | . | 0.30 | 0.17 | 0.20 | 0.26 | 0.07 | 0.12 | 0.07 | 0.19 | 0.12 | 0.31 | 0.17 | 0.14 | 0.751 |
| send 3 | 0.17 | 0.02 | 0.15 | 0.22 | 0.26 | 0.24 | 0.11 | 0.10 | 0.02 | 0.14 | 0.26 | 0.33 | 0.14 | 0.10 | 0.479 |
| send 4 | 0.33 | . | 0.04 | 0.22 | 0.28 | 0.37 | 0.09 | 0.31 | 0.02 | 0.02 | 0.17 | 0.48 | 0.19 | 0.12 | 0.550 |
| send 5 | 0.61 | . | . | 0.13 | 0.19 | 0.30 | 0.39 | 0.48 | . | 0.02 | 0.10 | 0.36 | 0.33 | 0.19 | 0.063 |
| send 6 | 0.38 | . | 0.06 | 0.19 | 0.28 | 0.28 | 0.20 | 0.33 | . | 0.12 | 0.12 | 0.36 | 0.21 | 0.19 | 0.586 |
| send 7 | 0.38 | . | 0.07 | 0.19 | 0.24 | 0.28 | 0.22 | 0.25 | 0.07 | 0.05 | 0.17 | 0.31 | 0.29 | 0.12 | 0.266 |
| send 8 | 0.31 | 0.04 | 0.07 | 0.15 | 0.31 | 0.24 | 0.19 | 0.29 | 0.05 | 0.07 | 0.12 | 0.40 | 0.21 | 0.14 | 0.669 |
| send 9 | 0.32 | 0.06 | 0.11 | 0.13 | 0.17 | 0.31 | 0.22 | 0.23 | 0.10 | 0.12 | 0.14 | 0.10 | 0.38 | 0.17 | 0.600 |
| send 10 | 0.28 | 0.06 | 0.15 | 0.11 | 0.19 | 0.26 | 0.24 | 0.36 | 0.10 | 0.02 | 0.10 | 0.26 | 0.33 | 0.19 | 0.803 |
|  |  |  |  |  |  |  |  |  |  |  |  |  |  |  |  |
| *Exp 2* | *N=71* | | | | | | | *N=72* | | | | | | |  |
| send 0 | -0.20 | 0.31 | 0.15 | 0.10 | 0.18 | 0.21 | 0.04 | -0.15 | 0.38 | 0.04 | 0.10 | 0.21 | 0.19 | 0.08 | 0.866 |
| send 1 | -0.12 | 0.24 | 0.18 | 0.11 | 0.20 | 0.17 | 0.10 | 0.02 | 0.21 | 0.14 | 0.07 | 0.22 | 0.26 | 0.10 | 0.265 |
| send 2 | -0.06 | 0.06 | 0.30 | 0.18 | 0.21 | 0.21 | 0.04 | 0.05 | 0.10 | 0.19 | 0.14 | 0.26 | 0.21 | 0.10 | 0.333 |
| send 3 | 0.15 | 0.04 | 0.07 | 0.28 | 0.31 | 0.21 | 0.08 | 0.12 | 0.04 | 0.07 | 0.33 | 0.25 | 0.21 | 0.10 | 0.830 |
| send 4 | 0.33 | 0.01 | 0.06 | 0.13 | 0.38 | 0.38 | 0.04 | 0.40 | . | 0.01 | 0.14 | 0.42 | 0.35 | 0.08 | 0.530 |
| send 5 | 0.59 | 0.01 | 0.03 | 0.07 | 0.23 | 0.27 | 0.39 | 0.67 | . | . | 0.07 | 0.22 | 0.28 | 0.43 | 0.483 |
| send 6 | 0.35 | 0.03 | 0.06 | 0.13 | 0.39 | 0.20 | 0.20 | 0.38 | . | 0.01 | 0.21 | 0.26 | 0.44 | 0.07 | 0.748 |
| send 7 | 0.21 | 0.04 | 0.14 | 0.17 | 0.23 | 0.30 | 0.13 | 0.17 | 0.03 | 0.11 | 0.25 | 0.29 | 0.19 | 0.13 | 0.578 |
| send 8 | 0.14 | 0.08 | 0.20 | 0.10 | 0.25 | 0.18 | 0.18 | 0.22 | 0.06 | 0.14 | 0.13 | 0.29 | 0.24 | 0.15 | 0.568 |
| send 9 | 0.16 | 0.11 | 0.14 | 0.13 | 0.21 | 0.20 | 0.21 | 0.10 | 0.15 | 0.14 | 0.06 | 0.32 | 0.24 | 0.10 | 0.363 |
| send 10 | 0.08 | 0.17 | 0.15 | 0.08 | 0.18 | 0.25 | 0.15 | 0.14 | 0.18 | 0.08 | 0.07 | 0.31 | 0.18 | 0.18 | 0.829 |
|  |  |  |  |  |  |  |  |  |  |  |  |  |  |  |  |
| *Pooled* | *N=125* | | | | | | | *N=114* | | | | | | |  |
| send 0 | -0.22 | 0.32 | 0.15 | 0.10 | 0.18 | 0.22 | 0.03 | -0.11 | 0.33 | 0.04 | 0.11 | 0.25 | 0.21 | 0.06 | 0.425 |
| send 1 | -0.11 | 0.23 | 0.18 | 0.12 | 0.18 | 0.21 | 0.08 | -0.00 | 0.18 | 0.16 | 0.10 | 0.25 | 0.23 | 0.09 | 0.281 |
| send 2 | -0.01 | 0.03 | 0.30 | 0.18 | 0.21 | 0.23 | 0.06 | 0.08 | 0.09 | 0.19 | 0.13 | 0.28 | 0.19 | 0.11 | 0.380 |
| send 3 | 0.15 | 0.03 | 0.10 | 0.26 | 0.29 | 0.22 | 0.10 | 0.11 | 0.04 | 0.10 | 0.31 | 0.28 | 0.18 | 0.10 | 0.544 |
| send 4 | 0.33 | 0.01 | 0.05 | 0.17 | 0.34 | 0.38 | 0.06 | 0.37 | 0.01 | 0.02 | 0.15 | 0.44 | 0.29 | 0.10 | 0.925 |
| send 5 | 0.60 | 0.01 | 0.02 | 0.10 | 0.21 | 0.28 | 0.39 | 0.60 | . | 0.01 | 0.08 | 0.27 | 0.30 | 0.34 | 0.602 |
| send 6 | 0.37 | 0.02 | 0.06 | 0.15 | 0.34 | 0.23 | 0.20 | 0.36 | . | 0.05 | 0.18 | 0.30 | 0.36 | 0.11 | 0.890 |
| send 7 | 0.28 | 0.02 | 0.11 | 0.18 | 0.23 | 0.29 | 0.17 | 0.20 | 0.04 | 0.09 | 0.22 | 0.30 | 0.23 | 0.12 | 0.206 |
| send 8 | 0.22 | 0.06 | 0.14 | 0.12 | 0.28 | 0.21 | 0.18 | 0.24 | 0.05 | 0.11 | 0.12 | 0.33 | 0.23 | 0.15 | 0.915 |
| send 9 | 0.23 | 0.09 | 0.13 | 0.13 | 0.19 | 0.25 | 0.22 | 0.15 | 0.13 | 0.13 | 0.09 | 0.24 | 0.29 | 0.12 | 0.222 |
| send 10 | 0.17 | 0.12 | 0.15 | 0.10 | 0.18 | 0.26 | 0.19 | 0.22 | 0.15 | 0.06 | 0.08 | 0.29 | 0.24 | 0.18 | 0.849 |

The table consists of three panels. The first shows data from the first experiment, the second shows data from the second, and the third presents pooled data. In each panel, the shares of participants that gave the respective rating for each amount sent by the dictator is reported. The modal response is highlighted. In addition, the mean appropriateness rating on a scale from -1 to 1 is given. The last column presents p-values of Mann-Whitney-U tests between the two treatments.

Table A6: Appropriateness ratings *Baseline* vs. *First*

|  | Baseline | | | | | | | First | | | | | | |  |
| --- | --- | --- | --- | --- | --- | --- | --- | --- | --- | --- | --- | --- | --- | --- | --- |
| action | mean | --- | -- | - | + | ++ | +++ | mean | --- | -- | - | + | ++ | +++ | p-value |
| *All data* | *N=203* | | | | | | | *N=204* | | | | | | |  |
| send 0 | -0.10 | 0.24 | 0.15 | 0.12 | 0.22 | 0.21 | 0.07 | -0.08 | 0.27 | 0.10 | 0.11 | 0.22 | 0.18 | 0.11 | 0.819 |
| send 1 | -0.00 | 0.15 | 0.18 | 0.15 | 0.19 | 0.24 | 0.09 | 0.03 | 0.13 | 0.17 | 0.15 | 0.24 | 0.20 | 0.11 | 0.723 |
| send 2 | 0.08 | 0.03 | 0.24 | 0.17 | 0.23 | 0.27 | 0.06 | 0.11 | 0.04 | 0.18 | 0.21 | 0.25 | 0.23 | 0.10 | 0.581 |
| send 3 | 0.20 | 0.02 | 0.09 | 0.26 | 0.27 | 0.27 | 0.10 | 0.20 | 0.02 | 0.11 | 0.22 | 0.28 | 0.27 | 0.09 | 0.996 |
| send 4 | 0.31 | 0.01 | 0.05 | 0.16 | 0.37 | 0.33 | 0.07 | 0.34 | 0.01 | 0.06 | 0.17 | 0.32 | 0.31 | 0.13 | 0.333 |
| send 5 | 0.56 | 0.01 | 0.01 | 0.10 | 0.24 | 0.30 | 0.33 | 0.60 | 0.01 | 0.02 | 0.06 | 0.25 | 0.3 | 0.35 | 0.531 |
| send 6 | 0.38 | 0.02 | 0.04 | 0.13 | 0.37 | 0.26 | 0.18 | 0.39 | 0.02 | 0.05 | 0.13 | 0.29 | 0.36 | 0.15 | 0.640 |
| send 7 | 0.30 | 0.02 | 0.1 | 0.16 | 0.26 | 0.32 | 0.14 | 0.38 | 0.00 | 0.07 | 0.14 | 0.32 | 0.27 | 0.19 | 0.255 |
| send 8 | 0.24 | 0.05 | 0.13 | 0.13 | 0.27 | 0.27 | 0.16 | 0.32 | 0.03 | 0.11 | 0.13 | 0.25 | 0.30 | 0.18 | 0.216 |
| send 9 | 0.26 | 0.07 | 0.11 | 0.12 | 0.24 | 0.29 | 0.17 | 0.24 | 0.08 | 0.10 | 0.14 | 0.24 | 0.24 | 0.20 | 0.891 |
| send 10 | 0.19 | 0.12 | 0.13 | 0.11 | 0.18 | 0.28 | 0.18 | 0.24 | 0.12 | 0.07 | 0.13 | 0.24 | 0.24 | 0.21 | 0.569 |
|  |  |  |  |  |  |  |  |  |  |  |  |  |  |  |  |
| *Understood incentives* | | | | *N=125* | | | | *N=120* | | | | | | |  |
| send 0 | -0.22 | 0.32 | 0.15 | 0.10 | 0.18 | 0.22 | 0.03 | -0.21 | 0.38 | 0.09 | 0.12 | 0.16 | 0.14 | 0.12 | 0.958 |
| send 1 | -0.11 | 0.23 | 0.18 | 0.12 | 0.18 | 0.21 | 0.08 | -0.15 | 0.18 | 0.24 | 0.17 | 0.17 | 0.13 | 0.10 | 0.781 |
| send 2 | -0.01 | 0.03 | 0.30 | 0.18 | 0.21 | 0.23 | 0.06 | -0.03 | 0.07 | 0.22 | 0.25 | 0.22 | 0.17 | 0.08 | 0.886 |
| send 3 | 0.15 | 0.03 | 0.10 | 0.26 | 0.29 | 0.22 | 0.10 | 0.11 | 0.02 | 0.15 | 0.26 | 0.30 | 0.19 | 0.08 | 0.453 |
| send 4 | 0.33 | 0.01 | 0.05 | 0.17 | 0.34 | 0.38 | 0.06 | 0.32 | 0.02 | 0.06 | 0.15 | 0.37 | 0.31 | 0.10 | 0.867 |
| send 5 | 0.60 | 0.01 | 0.02 | 0.10 | 0.21 | 0.28 | 0.39 | 0.63 | 0.02 | 0.01 | 0.05 | 0.24 | 0.28 | 0.41 | 0.671 |
| send 6 | 0.37 | 0.02 | 0.06 | 0.15 | 0.34 | 0.23 | 0.20 | 0.43 | 0.03 | 0.03 | 0.12 | 0.29 | 0.34 | 0.19 | 0.256 |
| send 7 | 0.28 | 0.02 | 0.11 | 0.18 | 0.23 | 0.29 | 0.17 | 0.39 | 0.01 | 0.07 | 0.13 | 0.30 | 0.28 | 0.20 | 0.238 |
| send 8 | 0.22 | 0.06 | 0.14 | 0.12 | 0.28 | 0.21 | 0.18 | 0.28 | 0.04 | 0.13 | 0.13 | 0.22 | 0.29 | 0.18 | 0.396 |
| send 9 | 0.23 | 0.09 | 0.13 | 0.13 | 0.19 | 0.25 | 0.22 | 0.22 | 0.09 | 0.11 | 0.15 | 0.23 | 0.20 | 0.23 | 0.873 |
| send 10 | 0.17 | 0.12 | 0.15 | 0.10 | 0.18 | 0.26 | 0.19 | 0.23 | 0.14 | 0.07 | 0.12 | 0.23 | 0.20 | 0.25 | 0.501 |

The table consists of two panels. The first shows all data and the second shows data from participants who correctly answered the question on understanding the incentives. In each panel, the shares of participants that gave the respective rating for each amount sent by the dictator is reported. The modal response is highlighted. In addition, the mean appropriateness rating on a scale from -1 to 1 is given. The last column presents p-values of Mann-Whitney-U tests between the two treatments.

Table A7: Appropriateness ratings *Baseline* vs. *Second*

|  | Baseline | | | | | | | Second | | | | | | |  |
| --- | --- | --- | --- | --- | --- | --- | --- | --- | --- | --- | --- | --- | --- | --- | --- |
| action | mean | --- | -- | - | + | ++ | +++ | mean | --- | -- | - | + | ++ | +++ | p-value |
| *All data* | *N=203* | | | | | | | *N=199* | | | | | | |  |
| send 0 | -0.10 | 0.24 | 0.15 | 0.12 | 0.22 | 0.21 | 0.07 | -0.11 | 0.28 | 0.12 | 0.12 | 0.21 | 0.19 | 0.10 | 0.866 |
| send 1 | -0.00 | 0.15 | 0.18 | 0.15 | 0.19 | 0.24 | 0.09 | 0.06 | 0.13 | 0.17 | 0.13 | 0.25 | 0.19 | 0.13 | 0.419 |
| send 2 | 0.08 | 0.03 | 0.24 | 0.17 | 0.23 | 0.27 | 0.06 | 0.08 | 0.07 | 0.15 | 0.21 | 0.27 | 0.23 | 0.08 | 0.964 |
| send 3 | 0.20 | 0.02 | 0.09 | 0.26 | 0.27 | 0.27 | 0.10 | 0.21 | 0.02 | 0.13 | 0.18 | 0.29 | 0.31 | 0.07 | 0.873 |
| send 4 | 0.31 | 0.01 | 0.05 | 0.16 | 0.37 | 0.33 | 0.07 | 0.33 | 0.01 | 0.05 | 0.16 | 0.40 | 0.31 | 0.09 | 0.768 |
| send 5 | 0.56 | 0.01 | 0.01 | 0.10 | 0.24 | 0.30 | 0.33 | 0.61 | 0.01 | 0.01 | 0.09 | 0.23 | 0.30 | 0.37 | 0.358 |
| send 6 | 0.38 | 0.02 | 0.04 | 0.13 | 0.37 | 0.26 | 0.18 | 0.43 | 0.01 | 0.06 | 0.09 | 0.35 | 0.34 | 0.16 | 0.346 |
| send 7 | 0.30 | 0.02 | 0.1 | 0.16 | 0.26 | 0.32 | 0.14 | 0.35 | 0.01 | 0.08 | 0.15 | 0.29 | 0.29 | 0.18 | 0.445 |
| send 8 | 0.24 | 0.05 | 0.13 | 0.13 | 0.27 | 0.27 | 0.16 | 0.30 | 0.04 | 0.11 | 0.15 | 0.24 | 0.27 | 0.20 | 0.313 |
| send 9 | 0.26 | 0.07 | 0.11 | 0.12 | 0.24 | 0.29 | 0.17 | 0.28 | 0.06 | 0.09 | 0.18 | 0.22 | 0.24 | 0.22 | 0.633 |
| send 10 | 0.19 | 0.12 | 0.13 | 0.11 | 0.18 | 0.28 | 0.18 | 0.23 | 0.14 | 0.08 | 0.12 | 0.21 | 0.26 | 0.21 | 0.579 |
|  |  |  |  |  |  |  |  |  |  |  |  |  |  |  |  |
| *Understood incentives* | | | *N=125* | | | | | *N=109* | | | | | | |  |
| send 0 | -0.22 | 0.32 | 0.15 | 0.10 | 0.18 | 0.22 | 0.03 | -0.29 | 0.37 | 0.15 | 0.12 | 0.16 | 0.16 | 0.06 | 0.417 |
| send 1 | -0.11 | 0.23 | 0.18 | 0.12 | 0.18 | 0.21 | 0.08 | -0.08 | 0.16 | 0.20 | 0.16 | 0.27 | 0.13 | 0.09 | 0.717 |
| send 2 | -0.01 | 0.03 | 0.30 | 0.18 | 0.21 | 0.23 | 0.06 | -0.00 | 0.07 | 0.19 | 0.22 | 0.26 | 0.21 | 0.05 | 0.937 |
| send 3 | 0.15 | 0.03 | 0.10 | 0.26 | 0.29 | 0.22 | 0.10 | 0.13 | 0.02 | 0.14 | 0.22 | 0.34 | 0.24 | 0.05 | 0.674 |
| send 4 | 0.33 | 0.01 | 0.05 | 0.17 | 0.34 | 0.38 | 0.06 | 0.35 | . | 0.06 | 0.15 | 0.38 | 0.33 | 0.09 | 0.876 |
| send 5 | 0.60 | 0.01 | 0.02 | 0.10 | 0.21 | 0.28 | 0.39 | 0.67 | . | . | 0.08 | 0.20 | 0.27 | 0.45 | 0.301 |
| send 6 | 0.37 | 0.02 | 0.06 | 0.15 | 0.34 | 0.23 | 0.20 | 0.48 | . | 0.02 | 0.10 | 0.35 | 0.38 | 0.16 | 0.158 |
| send 7 | 0.28 | 0.02 | 0.11 | 0.18 | 0.23 | 0.29 | 0.17 | 0.37 | 0.01 | 0.08 | 0.15 | 0.29 | 0.25 | 0.22 | 0.312 |
| send 8 | 0.22 | 0.06 | 0.14 | 0.12 | 0.28 | 0.21 | 0.18 | 0.24 | 0.05 | 0.15 | 0.17 | 0.18 | 0.24 | 0.22 | 0.566 |
| send 9 | 0.23 | 0.09 | 0.13 | 0.13 | 0.19 | 0.25 | 0.22 | 0.24 | 0.06 | 0.13 | 0.17 | 0.20 | 0.17 | 0.27 | 0.793 |
| send 10 | 0.17 | 0.12 | 0.15 | 0.10 | 0.18 | 0.26 | 0.19 | 0.17 | 0.19 | 0.07 | 0.10 | 0.17 | 0.20 | 0.26 | 0.832 |

The table consists of two panels. The first shows all data and the second shows data from participants who correctly answered the question on understanding the incentives. In each panel, the shares of participants that gave the respective rating for each amount sent by the dictator is reported. The modal response is highlighted. In addition, the mean appropriateness rating on a scale from -1 to 1 is given. The last column presents p-values of Mann-Whitney-U tests between the two treatments.
